# Household food insecurity, nutrient intakes and BMI in New Zealand infants

**DOI:** 10.1017/S1368980025101377

**Published:** 2025-11-03

**Authors:** Ioanna Katiforis, Claire Smith, Jillian J. Haszard, Sara E. Styles, Claudia Leong, Elizabeth A. Fleming, Rachael W. Taylor, Cathryn A. Conlon, Kathryn L. Beck, Pamela R. von Hurst, Lisa A. Te Morenga, Lisa Daniels, Madeleine Rowan, Maria Casale, Neve H. McLean, Alice M. Cox, Emily A. Jones, Kimberley J. Brown, Bailey R. Bruckner, Rosario Jupiterwala, Andrea Wei, Anne-Louise M. Heath

**Affiliations:** 1 Department of Human Nutrition, University of Otago, Dunedin 9054, New Zealand; 2 Haszard Biostatistics, Otago 9271, New Zealand; 3 Department of Applied Science and Social Practice, Ara Institute of Canterbury, Christchurch 8011, New Zealand; 4 Department of Medicine, University of Otago, Dunedin 9054, New Zealand; 5 School of Sport, Exercise and Nutrition, Massey University, Auckland 0745, New Zealand; 6 Research Centre for Hauora and Health, Massey University, Wellington 6140, New Zealand

**Keywords:** Body mass index, Dietary intake, Food insecurity, Infant, Nutrition, Public health

## Abstract

**Objective:**

The first year of life is a critical period when nutrient intakes can affect long-term health outcomes. Although household food insecurity may result in inadequate nutrient intakes or a higher risk of obesity, no studies have comprehensively assessed nutrient intakes of infants from food insecure households. This study aimed to investigate how infant nutrient intakes and BMI differ by household food security.

**Design:**

Cross-sectional analysis of the First Foods New Zealand study of infants aged 7–10 months. Two 24-h diet recalls assessed nutrient intakes. ‘Usual’ intakes were calculated using the multiple source method. BMI z-scores were calculated using WHO Child Growth Standards.

**Setting:**

Dunedin and Auckland, New Zealand.

**Participants:**

Households with infants (*n* 604) classified as: severely food insecure, moderately food insecure or food secure.

**Results:**

Nutrient intakes of food insecure and food secure infants were similar, aside from slightly higher free and added sugars intakes in food insecure infants. Energy intakes were adequate, and intakes of most nutrients investigated were likely to be adequate. Severely food insecure infants had a higher mean BMI z-score than food secure infants, although no significant differences in weight categories (underweight, healthy weight and overweight) were observed between groups.

**Conclusions:**

Household food insecurity, in the short term, does not appear to adversely impact the nutrient intakes and weight status of infants. However, mothers may be protecting their infants from potential nutritional impacts of food insecurity. Future research should investigate how food insecurity affects nutrient intakes of the entire household.

Household food insecurity, defined in New Zealand as a ‘limited or uncertain availability of nutritionally adequate and safe foods or limited ability to acquire acceptable foods in a socially acceptable way’,^(1)^ is a persistent public health issue. In 2015/2016, 17 % of New Zealand young children (0–4 years of age) lived in moderately to severely food insecure households^(1)^. These numbers have worsened in line with global economic disruption caused by the recent COVID-19 pandemic, which has severely impacted household incomes, levels of employment and widened existing socioeconomic inequalities^(2)^. In 2020/2022, 25 % of New Zealand families with infants experienced household food insecurity^(3)^. Similarly high prevalences of 15 % in the United Kingdom (UK) and 18 % in USA have been reported for households with children in the past 2 years^(4,5)^.

The WHO recommends infants consume a diverse diet comprising of healthy foods to decrease the risk of nutrient deficiencies, which may have long-term health impacts^(6)^. Adequate nutrition during infancy is essential for continued health in childhood^(7)^; however, infants living in food insecure households may be at risk of inadequate nutrition.

Food insecurity is associated with poorer dietary intakes^(8)^. Low consumption of fruits and vegetables, coupled with high exposure to energy-dense, nutrient-poor foods, has been reported in New Zealand infants living in households with food hardship^(9)^. These findings are especially concerning given that from 6 months of age, infants require nutrient-dense complementary foods to promote optimal growth and development^(6)^. Our previous research showed that infants in severely food insecure households were significantly more likely to frequently consume commercial baby food pouches than infants from more food secure households^(3)^, however, the corresponding impact on nutrient intakes is not known.

High intakes of added sugars^(10)^ and Na^(11)^ have been reported in *children* living in food insecure households. These reports are worrying as excessive consumption of either nutrient may contribute to hypertension in childhood^(12)^, with high sugars intakes also contributing to the development of dental caries and child obesity^(13)^. The evidence on nutrient intakes of *infants* in food insecure households in high-income countries is limited, with only one US study investigating nutrient intakes of infants from low-income households. This investigation was part of a larger study investigating factors related to blood lead levels and was conducted more than 20 years ago^(14)^. It is generally believed that adults protect children from the impacts of household food insecurity. However, as households experience varying levels of food insecurity, it is important to examine whether this is true for infants from households facing more severe food insecurity.

Food insecurity also has implications for body weight in young children. Childhood overweight and obesity follow a socio-economic gradient, with data from 2015/2016 showing that New Zealand young children 2–4 years of age who lived in food insecure households were significantly more likely to be overweight or obese than those in food secure households^(1)^. Whether the same applies in infancy is unknown, due to a scarcity of data. While reports from the US suggest that infants in food insecure households have higher BMI z-scores and a greater risk of overweight^(15,16)^, this finding is not universal,^(17)^ and comparable research does not appear to have been undertaken in other high-income countries. Further investigation of food insecurity and body weight in infants is warranted, as excess weight in infancy is associated with an increased risk of childhood obesity, which may lead to cardiovascular and metabolic disease in adulthood^(18)^.

In view of the importance to infant health of adequate nutrition and healthy weight status, there is a need to assess the nutrient intakes and BMI of infants living in food insecure households. Therefore, the aim of this study was to investigate how nutrient intakes and BMI differ by household food security in New Zealand infants.

## Methods

### Study design

Data were obtained from the cross-sectional, multi-centred First Foods New Zealand (FFNZ) study, which examined infant nutrition and health in a total of 625 infants from 7 to 10 months of age in Dunedin and Auckland, New Zealand. The current study is a secondary analysis of data from the FFNZ study. The sample size for the current study was determined by the number of participants required for the FFNZ primary outcomes^(19)^. As detailed methods for FFNZ are available in a published protocol paper^(19)^, only the methods relevant to the current study are described here.

### Participants and recruitment

Infant and primary-caregiver pairs were recruited between July 2020 and February 2022 via word-of-mouth and advertising in community settings regularly attended by families, such as parent group meetings. Efforts were made to recruit a similar proportion of families with young children living in areas of high socio-economic deprivation to that found in the New Zealand population^(20)^.

Primary caregivers who were 16 years of age or older and able to communicate in English were eligible to take part when their infant was 7·0–9·9 months of age. Infants who had recently participated in a nutrition intervention study were not eligible to participate as this may have changed the way they were fed. Participants attended 3–5 appointments (depending on whether they were part of a breastfeeding subsample) and received a NZ$150 supermarket voucher at the completion of the study. Written informed consent was obtained from adult participants (for themselves and on behalf of their infant) at the first appointment.

### Demographic characteristics

Demographic characteristics were collected using an online questionnaire at the first study visit. Age, sex, ethnicity and gestational status were collected for infants. For adult participants, age, ethnicity, highest level of education, employment status and maternal parity (for participants who were mothers of the infants) were collected. Adult participants were asked to specify the number of adults and children regularly living in the household and whether the infant received formal childcare outside the home.

For participants who identified themselves or their infant as having two or more ethnicities, ethnicity was prioritised into one ethnic group using the following order aligned with New Zealand Census categories: Māori, Pacific, Asian, New Zealand and other European, Other^(21)^. Area-level socio-economic deprivation was determined using the adult participant’s home address and described using the New Zealand Index of Deprivation 2018 deciles^(22)^. The deciles are constructed from a socio-economic deprivation score reflecting the extent of material and social deprivation in the area^(22)^.

### Household food insecurity

Household food security status was measured using the interviewer-administered ‘food security measurement tool for New Zealand households’^(23)^. This validated measure of household food security status in the New Zealand population comprises eight food insecurity indicator statements reflecting experiences of household financial constraint over the 12 months prior to administration of the questionnaire^(23)^. A validated total scoring method^(3)^ was used to score the responses and classify households into one of three categories of food insecurity (severely food insecure, moderately food insecure and food secure). ‘Severe food insecurity’ is characterised by affirmative responses to most of the indicator statements, including running out of food in the household, reducing food intake due to financial constraints and relying on assistance such as food grants or food banks (which is widely regarded as socially unacceptable in New Zealand^(23)^). ‘Moderate food insecurity’ reflects some household food insecurity, though not severe, and often involves purchasing a limited variety of foods, experiencing stress as a result of not having enough money for food, or stress as a result of being unable to provide desired foods for social occasions. In contrast, ‘food secure’ generally signifies the absence of these indicators in the household^(23)^.

### Infant anthropometric assessment

Infant length and weight were measured by trained researchers using calibrated equipment and procedures consistent with WHO anthropometric protocols^(24)^. A 99 cm measuring mat (model SE210; Seca) was used to measure infant length, and an electronic scale (models 334 and 345; Seca) for infant weight. If the first two recorded measurements differed by more than 0·7 cm in length, or 0·1 kg in weight, a third measurement was obtained and the mean of the two closest measurements recorded as the infant’s length or weight. Infant BMI was calculated (weight in kilograms divided by the length in metres squared). BMI z-scores were calculated using WHO Child Growth Standards^(25)^ and the ‘Zanthro’ package in Stata SE version 17.0 (StataCorp) statistical software.

### Dietary assessment

Two 24-h diet recalls were collected face-to-face by trained researchers with nutrition or dietetic backgrounds. Recalls were collected approximately a week apart on non-consecutive days and covered a 24-h duration (from midnight to midnight) over the previous day. To aid recall, participants were asked to take photographs on their mobile phone, or another camera device, of all foods and liquids offered to the infant in the relevant 24-h period. Moreover, participants were encouraged to refer to a set of portion size measurement aids during the interview.

Diet recalls were administered by researchers using a three-pass method. First, a ‘quick’ list was generated, where participants were asked to chronologically recall all foods and drinks (including breastfeeding and infant formula feeding occasions) offered to the infant on the day prior to the interview. Next, a detailed list captured information including times, durations and locations of eating occasions, descriptions of foods and drinks (such as food names and brands, ingredients and cooking methods for cooked recipes). Finally, the detailed list was reviewed with participants, and forgotten foods and drinks were probed for.

If infants were cared for by an adult (other than the adult participant) during the 24-h diet recall period, the participant was given a diary for the other caregiver to record all foods and drinks consumed by the infant while in their care, ensuring complete data on food intake were captured.

### Dietary supplement use

Data on dietary supplement use were collected via a self-administered questionnaire. Usual daily nutrient intakes from supplements were calculated from information on the identified product label by multiplying the nutrient content per unit with the frequency of supplement use within the past month (as reported by the participant).

### 24-h diet recall entry

Diet recalls were entered into FoodWorks 10 Professional (Xyris Software, Brisbane, Australia) for nutrient analysis. Nutrient data were sourced from FOODfiles 2018 Version 01 – the reference food composition database for New Zealand^(26)^. Composition data for commercial infant foods and infant formulas were generated separately^(27)^, as FOODfiles 2018 does not contain composition data for these products. FOODfiles defines ‘added sugars’ according to the definition of the US Food and Drug Administration: sugars (free, monosaccharides and disaccharides), sugars from honey and syrups and sugars from concentrated fruit and vegetable juices that are in excess of what would be expected from the same volume of 100 % fruit or vegetable juice of the same type^(28)^. Data entered into FoodWorks were checked for accuracy and consistency following a dietary data entry protocol.

### Breast milk intake and nutrient intake from breast milk

Breast milk intake was calculated in a subsample of infants (*n* 157) using the stable isotope deuterium oxide ‘dose-to-mother’ technique^(29)^. Briefly, this technique requires administration of an oral dose of deuterium oxide to the breastfeeding mother following a baseline saliva sample collection and then three post-dose saliva samples from the mother and her breastfed infant over 14 d to measure the enrichment of deuterium oxide in their saliva^(29)^.

Breast milk intake constitutes a large portion of some infant diets and therefore should be estimated as accurately as possible. However, accurate estimation of human milk intake is challenging, and current methods are either highly inaccurate with no, or almost no, variation (e.g. one-size-fits-all methods used by other large-scale studies)^(30)^, have a high respondent burden that may influence intake (e.g. test weighing) or are restrictively expensive (e.g. dose-to-mother deuterium oxide stable isotope method). Using the measurements from our dose-to-mother subsample, we developed an equation to estimate usual breast milk intake using prediction modelling techniques that is significantly more accurate and easier to administer than previous methods^(31)^. This equation uses demographic characteristics, energy intake from complementary foods, number of breastfeeds per day and infant formula intake to estimate usual human milk intake.

For breastfed infants who were not part of the subsample, the predictive equation was applied to estimate their usual daily breast milk intake^(31)^. This intake was multiplied by the nutrient composition of breast milk (determined by laboratory analysis of breast milk samples obtained in the subsample) to calculate nutrient intake from breast milk.

### Calculation of usual nutrient intakes

‘Usual’ daily intakes of nutrients from complementary food and drinks reported in the 24-h diet recalls were calculated using the multiple source method^(32)^. These were added to usual daily nutrient intakes from infant formula, breast milk and dietary supplements to estimate usual nutrient intakes from the total diet.

### Statistical analysis

Stata SE, version 17.0 (StataCorp) statistical software was used to analyse data, with *P* < 0·05 considered to indicate statistical significance.

Demographic characteristics of the overall sample and by household food security status were summarised using descriptive statistics. Linear regression analysis was used to estimate mean differences (with 95 % CI) in energy and nutrient intakes among infants living in moderately or severely food insecure households, compared with those in food secure households.

Usual daily total energy intakes were compared with estimated energy requirements for infants at 8 months of age^(33)^. Adequacy of Fe intake was determined using the full probability approach, and adequacy of Zn intake was determined using the estimated average requirement (EAR) cut-point method^(34)^. For other nutrients, none of which have an EAR for New Zealand and Australia^(34)^, and mean group intakes were compared with the adequate intake (AI) to determine the likelihood of adequacy. The adequacy of nutrient intakes by household food security status was summarised using descriptive statistics.

Mean differences (and 95 % CI) in BMI z-scores of moderately food insecure or severely food insecure infants, compared with food secure infants, were estimated using linear regression analysis. Infants with BMI z-scores of < −2sd were classified as ‘underweight’, those with BMI z-scores between −2sd and +2sd were classified as ‘healthy weight’ and those with a BMI z-score > +2sd were classified as ‘overweight’^(35)^.

Both unadjusted and adjusted models were presented for energy and nutrient intakes and BMI z-scores. Adjusted models included caregiver age, caregiver education, caregiver ethnicity and number of children in the household (selected *a priori*).

## Results

### Participant demographics

**Figure 1:**
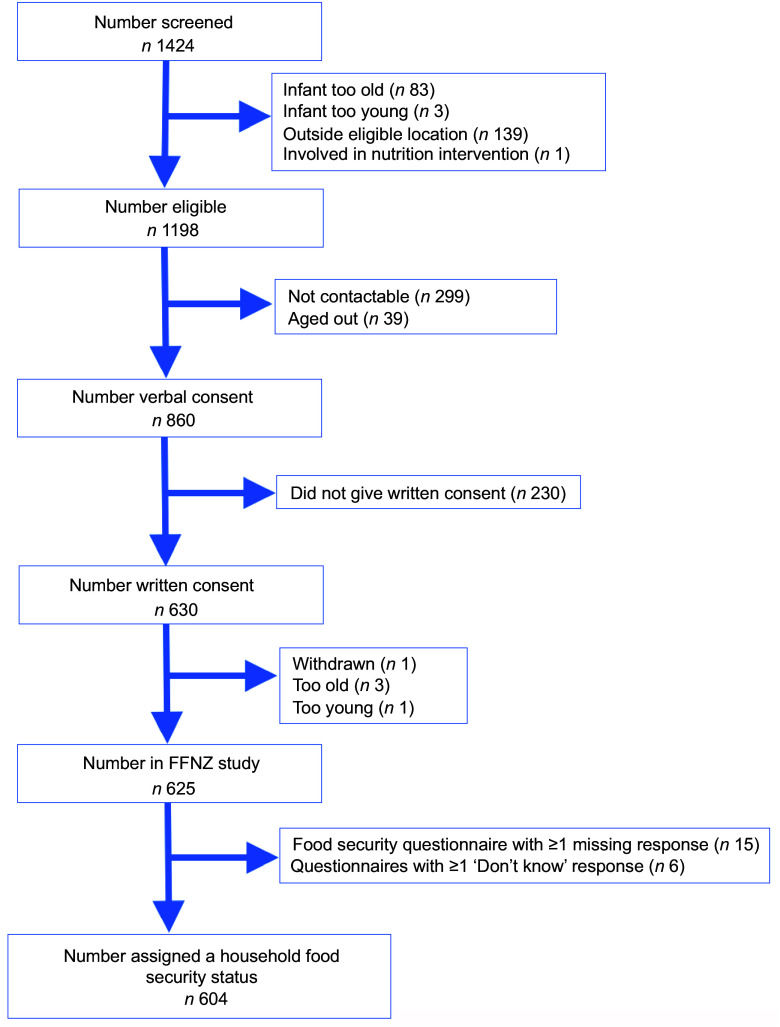
FFNZ study recruitment flowchart. FFNZ, First Foods New Zealand.

Details of the recruitment of the final sample of 604 participants are available in a study recruitment flow chart (Figure 1).

Table 1 describes the participant demographic characteristics. At the time of study participation, infants had a mean (sd) age of 8·4 (0·8) months. Approximately half the infants were female (46 %). The sample was ethnically diverse, with 21 % of infants identified by their caregiver as Māori, 15 % Asian and 7 % Pacific, compared with 60 % who were identified as New Zealand or other European.

**Table 1.** Demographic characteristics for all participants and by household food security status (*n* 604)

			Household food security status^*^						
Characteristic	Full sample (*n* 604)		Food secure^†^ (*n* 453)		Moderately food insecure (*n* 105)		Severely food insecure (*n* 46)		*P*-value
	Mean	sd	Mean	sd	Mean	sd	Mean	sd	
Infant characteristics									
Age (months), mean (sd)	8·4	0·8	8·4	0·8	8·3	0·8	8·3	1·0	0·241
	*n*	%	*n*	%	*n*	%	*n*	%	
Sex^‡^									0·570
Male	326	54·0	244	53·8	60	57·1	22	47·8	
Female	277	45·9	208	45·9	45	42·9	24	52·2	
Rather not say	1	0·2	1	0·2	0	0·0	0	0·0	
	Mean	sd	Mean	sd	Mean	sd	Mean	sd	
Length (cm), mean (sd)^§^	70·66	3·04	70·69	2·90	70·51	3·09	70·73	4·15	0·859
Weight (kg), mean (sd)^||^	8·77	1·15	8·75	1·08	8·78	1·31	8·96	1·40	0·495
BMI z-score, mean (sd)^¶^	0·29	1·00	0·26	0·97	0·31	1·15	0·53	0·90	**0·048**
WFL z-score, mean (sd)	0·38	0·99	0·35	0·96	0·40	1·14	0·65	0·91	0·174
	*n*	%	*n*	%	*n*	%	*n*	%	
Ethnicity									**< 0·001**
Māori	124	20·5	69	15·2	33	31·4	22	47·8	
Pacific	41	6·8	25	5·5	9	8·6	7	15·2	
Asian	88	14·6	76	16·8	10	9·5	2	4·4	
Other^***^	16	2·7	11	2·4	3	2·9	2	4·4	
New Zealand and other European^†††^	335	55·5	272	60·0	50	47·6	13	28·3	
Gestational status^**^									**0·032**
Pre-term (< 37 weeks)	44	7·3	30	6·6	6	5·7	8	17·8	
Term (≥ 37 weeks)	559	92·7	423	93·4	99	94·3	37	82·2	
Caregiver characteristics									
	Mean	sd	Mean	sd	Mean	sd	Mean	sd	
Age (years), mean (sd)^††^	32·7	4·8	33·4	4·3	31·2	5·6	30·1	6·4	**< 0·001**
	*n*	%	*n*	%	*n*	%	*n*	%	
Age group (years)^‡‡^									**< 0·001**
< 25	46	7·6	14	3·1	20	19·1	12	26·1	
25–< 35	359	59·6	281	62·3	57	54·3	21	45·7	
≥ 35	197	32·7	156	34·6	28	26·7	13	28·3	
Relation to infant									–^‡‡‡^
Mother	596	98·7	447	98·7	104	99·1	45	97·8	
Maternal parity^§§,§§§^									**< 0·001**
One	289	48·0	233	51·4	47	45·2	9	19·6	
Two	187	31·0	142	31·4	31	29·8	14	30·4	
Three	85	14·1	59	13·0	16	15·4	10	21·7	
Four or more	42	7·0	19	4·2	10	9·6	13	28·3	
Ethnicity									**< 0·001**
Māori	80	13·3	46	10·2	19	18·1	15	32·6	
Pacific	30	5·0	18	4·0	5	4·8	7	15·2	
Asian	83	13·7	71	15·7	12	11·4	0	0·0	
Other^***^	15	2·5	10	2·2	4	3·8	1	2·2	
New Zealand and other European^†††^	396	65·6	308	68·0	65	61·9	23	50·0	
Highest level of education^||||^									**< 0·001**
School (primary or secondary)	92	15·3	52	11·5	23	21·9	17	37·8	
Polytechnic or similar tertiary institution^||||||^	121	20·1	69	15·2	34	32·4	18	40·0	
University	390	64·7	332	73·3	48	45·7	10	22·2	
Current employment status									**< 0·001**
Not employed	183	30·3	111	24·5	38	36·2	34	73·9	
Employed part-time	131	21·7	101	22·3	25	23·8	5	10·9	
Employed full-time	70	11·6	53	11·7	14	13·3	3	6·5	
Paid parental leave	58	9·6	48	10·6	9	8·6	1	2·2	
Unpaid parental leave	162	26·8	140	30·9	19	18·1	3	6·5	
Household characteristics									
Childcare outside the home^¶¶¶^									0·105
No	498	82·5	375	82·8	81	77·1	42	91·3	
Yes	106	17·6	78	17·2	24	22·9	4	8·7	
Number of children in household^¶¶,****^									**< 0·001**
One	272	45·1	223	49·3	41	39·1	8	17·4	
Two	194	32·2	147	32·5	34	32·4	13	28·3	
Three	92	15·3	63	13·9	18	17·1	11	23·9	
Four or more	45	7·5	19	4·2	12	11·4	14	30·4	
Number of adults in household^††††^									**< 0·001**
One	23	3·8	9	2·0	4	3·8	10	21·7	
Two	502	83·1	385	85·0	89	84·8	28	60·9	
Three	40	6·6	29	6·4	7	6·7	4	8·7	
Four or more	39	6·5	30	6·6	5	4·8	4	8·7	
Socio-economic deprivation decile									**< 0·001**
1–3 (Low)	173	28·6	147	32·5	18	17·1	8	17·4	
4–7	271	44·9	207	45·7	46	43·8	18	39·1	
8–10 (High)	160	26·5	99	21·9	41	39·1	20	43·5	

Bold text indicates a statistically significant difference (*P* < 0·05).

cm, centimetres; kg, kilograms; sd, standard deviation; WFL, weight for length.

* ‘Food secure’ is the reference category for all statistical comparisons.

† Percentages of the full sample and household food security status are reported vertically (i.e. by column). Percentages may not total to 100 due to rounding.

‡ ‘Rather not say’ excluded from statistical test.

§ *n* 6 missing data. ^||^ *n* 8 missing data. ^¶^ *n* 14 missing data. **n 1 missing datum. ^††^ *n* 2 missing data. ^‡‡^ *n* 2 missing data. ^§§^ *n* 1 missing datum. ^||||^ *n* 1 missing datum. ^¶¶^ *n* 1 missing datum.

*** ‘Other’ comprises Aboriginal, African, Native American, South American and West Asian.

††† ‘European’ comprises American, Australian, Canadian, European, New Zealand European and South African European.

‡‡‡ Unable to perform statistical test due to small numbers in cells.

§§§ Parity of the infant’s mother, who may not be the participant in the sample.

|||||| ‘Polytechnic’ may include bachelor’s degrees.

¶¶¶ Defined as the infant being ‘regularly looked after by someone other’ than the participant. Includes early childhood centre or home-based care.

**** Number of children who ‘usually (at least half the time)’ live in the household. Age of ‘child’ not defined, therefore may include adult children.

†††† Number of adults who ‘usually live’ in the household. Age of ‘adult’ not defined.

More than half (59 %) of caregivers were between 25 and 35 years of age, and almost all (99 %) caregivers were the mother of the infant in the study. For half the mothers (48 %), the infant was their first child. Overall, the sample was highly educated with two-thirds (65 %) of caregivers having a university education, compared with one-third (35 %) in the New Zealand adult population^(36)^. The levels of area-based socio-economic deprivation reflected those in the New Zealand population with 27 % of caregivers living in an area of high socio-economic deprivation (deciles 8–10), compared with 29 % in the population of New Zealand households with young children^(20)^. Most infants lived in a two-adult household (83 %).

There were several differences in demographic characteristics by household food security status. Compared with food secure caregivers, caregivers experiencing food insecurity were more likely to be younger or of Māori or Pacific ethnicity, less likely to have a university education or current employment and more likely to live in sole-parent households and have a larger number of children in the household.

### Infant nutrient intakes

Several significant differences were observed in the nutrient intakes of infants living in food insecure households compared with those in food secure households with infants in moderately and severely food insecure households having higher free sugars, added sugars and Ca intakes and infants in moderately food insecure households having higher Fe, Zn, vitamin B_12_ and vitamin C intakes (Table 2). Adjustment for confounders attenuated most of these differences.

**Table 2.** Energy and nutrient intakes of infants by household food security status (*n* 604)^*^

	Mean intakes						Mean differences from food secure (95 % CI)							
Nutrient	Food secure† (*n* 453)		Moderately food insecure (*n* 105)		Severely food insecure (*n* 46)		Moderately food insecure				Severely food insecure			
							Unadjusted		Adjusted^‡^		Unadjusted		Adjusted^‡^	
	Mean	sd	Mean	sd	Mean	sd	Mean	95 % CI	Mean	95 % CI	Mean	95 % CI	Mean	95 % CI
Total energy														
kJ/d	3397	603	3426	591	3453	645	29	–99, 158	−35	–168, 97	56	–128, 240	−92	–293, 109
Macronutrients														
Protein														
g/d	22·4	7·6	22·9	6·8	22·4	6·6	0·4	–1·1, 2·0	0·4	–1·2, 2·0	0·0	–2·2, 2·2	−0·3	–2·8, 2·1
%TE	10·9	2·5	11·1	2·3	10·8	2·3	0·2	–0·3, 0·7	0·3	–0·2, 0·9	−0·1	–0·9, 0·6	0·1	–0·7, 1·0
Total fat														
g/d	38·5	8·0	37·9	7·5	38·1	7·6	−0·6	–2·3, 1·0	−0·6	–2·4, 1·1	−0·4	–2·8, 2·0	−0·2	–2·8, 2·5
%TE	43·0	6·0	41·8	5·0	42·0	5·8	−1·1	–2·4, 0·1	−0·4	–1·6, 0·9	−1·0	–2·8, 0·8	1·0	–0·9, 2·9
Total carbohydrate														
g/d	90·1	19·0	92·7	18·1	95·0	22·5	2·7	–1·4, 6·7	−1·1	–5·2, 3·0	4·9	–0·9, 10·8	−4·0	–10·2, 2·2
%TE	44·3	5·1	45·2	4·0	45·8	4·9	0·9	–0·2, 1·9	0·0	–1·1, 1·0	1·5	0·0, 3·0	−0·8	–2·4, 0·8
Total sugars														
g/d	63·3	13·6	62·5	15·5	68·5	14·7	−0·8	–3·8, 2·2	−2·7	–5·8, 0·4	**5·3**	**1·0, 9·5**	1·7	–3·0, 6·4
%TE	31·6	6·3	30·9	6·9	33·4	5·3	−0·7	–2·0, 0·7	−0·9	–2·3, 0·6	1·9	0·0, 3·8	1·8	–0·4, 3·9
Free sugars														
g/d	2·1	3·5	3·9	4·5	5·4	6·6	**1·7**	**0·9, 2·6**	**1·0**	**0·2, 1·9**	**3·3**	**2·0, 4·5**	**1·4**	**0·1, 2·7**
%TE	1·0	1·5	1·8	2·0	2·5	2·8	**0·8**	**0·5, 1·2**	**0·5**	**0·2, 0·9**	**1·5**	**1·0, 2·0**	**0·7**	**0·2, 1·3**
Added sugars														
g/d	1·8	3·1	3·1	3·8	4·5	5·3	**1·3**	**0·6, 2·0**	0·7	0·0, 1·4	**2·8**	**1·7, 3·8**	**1·2**	**0·1, 2·3**
%TE	0·8	1·3	1·4	1·7	2·1	2·3	**0·6**	**0·3, 0·9**	**0·4**	**0·1, 0·7**	**1·3**	**0·8, 1·7**	**0·7**	**0·2, 1·1**
Dietary fibre														
g/d	7·8	4·0	8·1	3·7	7·2	3·8	0·3	–0·5, 1·1	0·1	–0·7, 1·0	−0·6	–1·8, 0·5	−1·3	–2·6, 0·0
Micronutrients														
Ca														
mg/d	429	216	492	222	503	239	**63**	**17, 110**	38	–10, 86	**74**	**8, 141**	18	–55, 91
Fe														
mg/d	5·8	4·3	6·9	4·8	6·4	4·7	**1·0**	**0·1, 2·0**	0·5	–0·5, 1·4	0·6	–0·8, 1·9	−0·9	–2·4, 0·5
Zn														
mg/d	3·7	2·4	4·4	2·4	4·1	2·6	**0·6**	**0·1, 1·2**	0·4	–0·1, 0·9	0·4	–0·3, 1·1	−0·2	–1·0, 0·6
Vitamin B_12_														
µg/d	1·4	1·4	5·5	38·4§	1·5	1·3	**0·4**	**0·1, 0·7** ^||^	0·3	0·0, 0·6^||^	0·0	–4·8, 4·9	−0·1	5·5, 5·3
Vitamin C														
mg/d	72·5	44·6	98·0	196·4^¶^	72·8	47·3	6·8	–2·8, 16·4^**^	3·9	–5·9, 13·7^**^	0·3	–27·5, 28·0	−3·7	–34·7, 27·3

g, grams; kJ, kilojoules; µg, micrograms; mg, milligrams; TE, total energy.

Bold text indicates a statistically significant difference (*P* < 0·05).

* Total intake from food sources and dietary supplements.

† ‘Food secure’ is the reference category for all statistical comparisons.

‡ Estimates adjusted for caregiver age, caregiver education, caregiver ethnicity and number of children in household.

§ Mean intake, excluding high-dose supplement value: 1·8 µg/d.

|| When the high-dose supplement value was included, the mean difference in intake from food secure infants was substantially larger (unadjusted, 4·1 µg/d (95 % CI (0·7, 7·5)); adjusted, 4·0 µg/d (95 % CI (0·4, 7·6))).

¶ Mean intake, excluding high-dose supplement value: 79·3 mg/d.

** When the high-dose supplement value was included, the mean difference in intake from food secure infants was substantially larger (unadjusted, 25·5 mg/d (95 % CI (6·0, 44·9)); adjusted, 24·4 mg/d (95 % CI (3·9, 44·8))).

Total sugars intakes were higher for infants in severely food insecure households than those in food secure households (5·3 g/d higher, 95 % CI (1·0, 9·5)), but after adjusting for confounders, this difference was attenuated to 1·7 g/d (–3·0, 6·4). No evidence of a difference in total sugars intakes in moderately food insecure infants compared with food secure infants was observed (adjusted, –2·7 g/d; 95 % CI (–5·8, 0·4)).

Added sugars intakes were higher in moderately food insecure infants (1·3 g/d higher, 95 % CI (0·6, 2·0)) and even higher for severely food insecure infants (2·8 g/d higher, 95 % CI (1·7, 3·8)) than for food secure infants. Results for free sugars were similar to those for added sugars.

Although the mean vitamin B_12_ intake of infants in moderately food insecure households was 5·5 µg/d; a higher intake than was observed in food secure infants (1·4 µg/d), this was because of consumption of a high dose supplement by a single infant. Removal of that value resulted in a mean intake of 1·8 µg/d for moderately food insecure infants, and the corresponding difference in mean vitamin B_12_ intake compared with food secure infants was attenuated to 0·3 µg/d (95 % CI (0·0, 0·6)). No significant differences were observed in the mean vitamin B_12_ intake of severely food insecure infants compared with food secure infants.

The mean vitamin C intake of moderately food insecure infants (98·0 mg/d) was significantly higher than for food secure infants (unadjusted difference, 25·5 mg/d; 95 % CI (6·0, 44·9)). However, this was again because of consumption of a high dose supplement by a single infant. Removal of that value resulted in a mean intake of 79·3 mg/d for moderately food insecure infants and attenuated the difference in vitamin C intake between moderately food insecure and food secure infants (unadjusted, 3·9 mg/d; 95 % CI (–5·9, 13·7)).

Ca intakes were higher for moderately food insecure (63 mg/d, 95 % CI (17, 110)) and severely food insecure (74 mg/d, 95 % CI (8, 141)) infants than for food secure infants; however, differences were attenuated upon adjustment for confounding variables.

The mean Fe intake of infants in moderately food insecure households (unadjusted difference, 1·0 mg/d; 95 % CI (0·1, 2·0)) was slightly higher than for food secure infants. However, no significant differences were observed in the mean Fe intake of severely food insecure infants compared with food secure infants (unadjusted, 0·6 mg/d; 95 % CI (–0·8, 1·9)). A similar pattern was observed for differences in Zn intakes (moderately food insecure: unadjusted, 0·6 mg/d; 95 % CI (0·1, 1·2), severely food insecure: unadjusted, 0·4 mg/d; 95 % CI (–0·3, 1·1)).

### Adequacy of infant nutrient intakes

Mean total energy intakes were similar in infants living in moderately or severely food insecure households, compared with those of food secure infants (Table 2), and were above the estimated energy requirements for boys and girls from 7 to 12 months of age (Table 3).

**Table 3.** Adequacy of infant nutrient intakes by household food security status (*n* 604)^*^,†

Nutrients	Nutrient reference values‡	Mean intakes					
		Food secure (*n* 453)		Moderately food insecure (*n* 105)		Severely food insecure (*n* 46)	
		Mean	sd	Mean	sd	Mean	sd
Total energy^§^							
kJ/d	2700–3000 kJ/d	3397	603	3426	591	3453	645
Nutrients with an EAR							
Fe							
Amount (mg/d)	7 mg/d	5·8	4·3	6·9	4·8	6·4	4·7
(%) inadequate^||^		%		%		%	
		61·7		50·7		54·7	
Zn (mg/d)							
Amount (mg/d)	2·5 mg/d	3·7	2·4	4·4	2·4	4·1	2·6
*n* (%) inadequate^¶^		*n*	%	*n*	%	*n*	%
		182	40·2	34	32·4	18	39·1

		Mean	sd	Mean	sd	Mean	sd
Nutrients with an AI^**^ ^,^ ^††^							
Protein (g/d)	14 g/d	22·4	7·6	22·9	6·8	22·4	6·6
Total fat (g/d)	30 g/d	38·5	8·0	37·9	7·5	38·1	7·6
Total carbohydrate (g/d)	95 g/d	**90·1**	**19·0**	**92·7**	**18·1**	95·0	22·5
Ca (mg/d)	270 mg/d	429	216	492	222	503	239
Vitamin B_12_ (µg/d)	0·5 µg/d	1·4	1·4	5·5	38·4	1·5	1·3
Vitamin C (mg/d)	30 mg/d	72·5	44·6	98·0	196·4	72·8	47·4

AI, adequate intake; EAR, estimated average requirement; g, grams; kJ, kilojoules, µg, micrograms; mg, milligrams.

Bold text indicates a statistically significant difference (*P* < 0·05).

* Total intake from food sources and dietary supplements.

† Data presented as mean (sd) unless indicated otherwise.

‡ Nutrient reference values for infants 7–12 months of age; estimated energy requirement for infants at 8 months of age^(33)^.

§ Range refers to estimated energy requirements for girls (2700 kJ/d) and boys (3000 kJ/d). Total energy intakes were not reported by sex, as the aim and analysis focused on adequacy of nutrient intakes across levels of household food insecurity rather than by sex.

|| Prevalence of inadequate Fe intake determined using the full probability approach^(34)^. This approach determines the probability of each observed value being inadequate and does not represent a number of participants, therefore ‘*n*’ is not reported.

¶ Prevalence of inadequate Zn intake estimated using the EAR cut-point method^(34)^.

** Group mean nutrient intakes at or above the AI are considered to indicate adequacy^(34)^.

†† An AI for dietary fibre has not been set because breast milk does not contain dietary fibre, and there are no functional criteria for dietary fibre in infants^(33)^.

The prevalence of inadequate Fe intakes was high in all three groups, ranging from half (50·6 %) of infants in moderately food insecure households to 61·7 % of infants in food secure households. The prevalence of inadequate Zn intakes followed a similar pattern, ranging from one-third (32·4 %) of moderately food insecure infants to 40·2 % of food secure infants.

Intakes of protein, total fat, Ca, vitamin B_12_ and vitamin C appeared to be sufficient in food secure and food insecure infants, with the mean intakes of these nutrients being higher than the AI. The likely adequacy of total carbohydrate intake could not be determined for food secure and moderately food insecure infants because the mean intake was below the AI of total carbohydrate.

### BMI z-score and weight status

Overall, infants had a mean (sd) BMI z-score of 0·29 (1·00). BMI z-score differed by household food security status (Table 1, *P* = 0·048), where mean BMI z-score tended to be higher in severely food insecure infants (0·53 (0·90)) compared with infants in food secure households (0·26 (0·97)). However, differences in mean BMI z-score between food insecurity categories were strongly attenuated after adjustment for confounding variables (moderately food insecure compared with food secure: 0·01, 95 % CI (–0·21, 0·23); severely food insecure compared with food secure: 0·15, 95 % CI (–0·19, 0·49)) (Table 4).

**Table 4.** Mean differences in infant BMI z-scores by household food security status (*n* 590)^*^

	BMI z-score						Mean differences from food secure (95 % CI)							
	Food secure^†^ (*n* 443)		Moderately food insecure (*n* 103)		Severely food insecure (*n* 44)		Moderately food insecure				Severely food insecure			
							Unadjusted		Adjusted‡		Unadjusted		Adjusted‡	
	Mean	sd	Mean	sd	Mean	sd	Mean	95 % CI	Mean	95 % CI	Mean	95 % CI	Mean	95 % CI
BMI z-score	0·26	0·97	0·31	1·15	0·53	0·90	0·05	–0·16, 0·27	0·01	–0·21, 0·23	0·27	–0·04, 0·58	0·15	–0·19, 0·49

* *n* 14 infants had missing data for BMI.

† ‘Food secure’ is the reference category for all statistical comparisons.

‡ Estimates adjusted for caregiver age, caregiver education, caregiver ethnicity and number of children in household.

**Figure 2:**
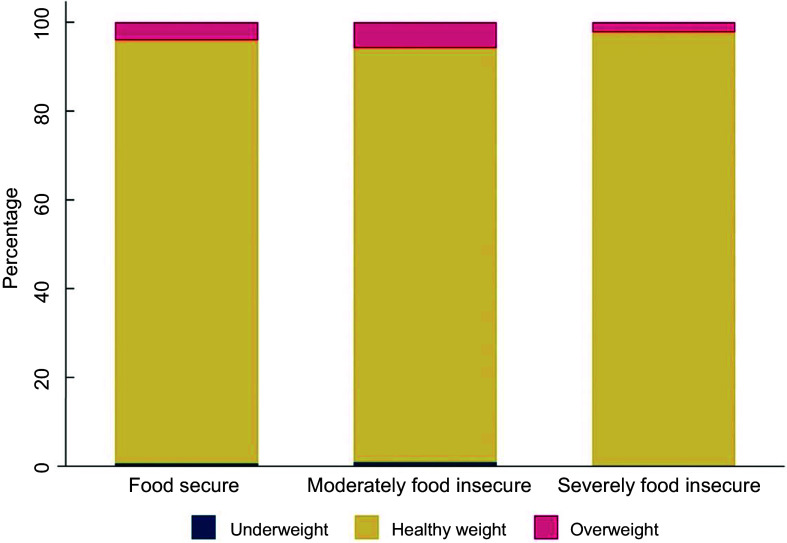
Bar chart showing weight status of infants by household food security status (*n* 590)^a b^. ^a^ *n* 14 infants had missing data for BMI. ^b^Categories defined as follows: Underweight: BMI z-score < −2sd, healthy weight: BMI z-score between −2sd and +2sd, overweight: BMI z-score > 2sd ^(35)^. Mean BMI z-score for sample: 0·29.

Almost all infants in the sample were of a healthy weight (food secure 95·3 %; moderately food insecure 93·2 %; severely food insecure 97·7 %) (Figure 2). The prevalence of overweight in the sample was low (food secure 4·1 %; moderately food insecure 5·8 %; severely food insecure 2·3 %), and the prevalence of underweight was even lower (≤ 1 % in each group).

## Discussion

To our knowledge, this study provides the first comprehensive investigation of nutrient intakes in infants living in food insecure households and the first investigation of BMI and household food insecurity in a non-US sample of infants.

Our findings suggest that living in a food insecure household did not adversely impact the nutrient intakes and weight status of infants. On average, food insecure infants did not have significantly different total energy intakes from food secure infants, and mean intakes in all three groups met the estimated energy requirements for infants. Although slightly higher mean intakes of free sugars and added sugars were observed in food insecure infants, the difference amounted to approximately one-quarter of a standard (5 ml) teaspoon, and intakes were low overall. Intakes of most key nutrients investigated (protein, total fat, Ca, vitamin B_12_ and vitamin C) appeared to be adequate. Inadequate Fe intakes were observed in at least half of the infants in the sample, and inadequate Zn intakes in more than 30 %, with the highest proportions of inadequate Fe and Zn intakes observed in food secure infants. Although the mean BMI z-score of severely food insecure infants was higher than that of food secure infants, weight status did not appear to be impacted. Proportions of infants with a healthy weight status were similar with over 90 % of infants in each group classified as being of a healthy weight.

The adequacy of energy intakes and likely adequacy of most macronutrient and micronutrient intakes assessed suggests that, overall, infants consumed adequate diets, regardless of whether they lived in a food insecure household. The only other study of nutrient intakes in food insecure infants that we are aware of is the study by Nolan *et al.* (2002). Unlike the current study, their assessment of energy and nutrient intakes in infants from low-income households in the US excluded infants who were breastfed, and the comparison of mean nutrient intakes with the RDA precludes investigation of the adequacy of the infants’ intakes^(14)^.

It is possible that the absence of evidence for lower nutrient intakes in food insecure infants compared with food secure infants in the current study was because mothers (who represented 99 % of participants) limited their own food intake to protect the infant’s diet from being compromised. It has been proposed that the lack of a clear association between food insecurity and poorer diet quality in children, unlike adults, may be a result of adults ‘buffering’ their children from food insecurity by altering their own diets during household food shortages^(8)^. However, it is also possible that, for infants, their largely milk-based diets help to protect them against inadequate nutrient intakes, so long as their access to breast milk or infant formula is not threatened. Maternal dietary intake and the contributions of breast milk and infant formula to infant nutrient intakes were not directly assessed in this study. Further investigation collecting these data is needed to clarify the role and impact of maternal buffering on the nutrient intakes of food insecure infants.

While New Zealand does not have a national food assistance program supporting food insecure or low-income families with infants, access to charitable community food support services or emergency financial assistance through government social welfare programs may have improved nutrient intakes in food insecure infants, particularly if mothers prioritised these resources for infant feeding. The high prevalence of inadequate Fe intakes in the sample overall is concerning and aligns with our observation that 23 % of the study infants had suboptimal Fe status^(37)^. While the highest prevalence of inadequate Fe intakes was observed in food secure infants (an unexpected finding given evidence of an association between child-level food insecurity and Fe deficiency anaemia in children < 36 months of age^(38)^), it is important to consider the infant milk source. Infant formula contains a higher concentration of Fe than breast milk^(39)^, and infant formulas sold in New Zealand must be fortified with Fe at a moderately high level (0·2 mg–0·5 mg/100 kJ of dry powder) to compensate for the lower bioavailability of Fe in infant formula^(40)^. *Post hoc* analyses revealed that severely food insecure infants were almost twice as likely to be consuming ≥ 2500 kJ/d total energy from infant formula than food secure infants (17·4 % *v*. 9·5 %, respectively, *P* = 0·060), and the mean (sd) Fe intake of infants consuming ≥ 2500 kJ/d total energy from infant formula was almost four times that of infants who did not consume infant formula (11·6 (2·4) *v*. 3·0 (2·9) mg/d). Therefore, the higher prevalence of inadequate Fe intake in food secure infants was likely a result of lower intakes of infant formula in food secure infants. This, however, is an argument for higher Fe intakes from complementary foods, such as Fe-fortified cereal^(41)^ rather than the use of infant formula, given the numerous benefits of breastfeeding for both infant and mother^(42)^.

Although we observed a high prevalence of inadequate Fe and Zn intakes across all three groups in our sample, public health nutrition strategies must prioritise improving intakes in food insecure families. This is particularly important given evidence that fewer food insecure young children aged 2–4 years in New Zealand meet healthy dietary indicators^(1)^. Introducing a targeted food subsidy program that provides pre-paid cards or cash transfers for meat, legumes, milk, fruits and vegetables to families with young children could improve Fe and Zn intakes in food insecure infants. Health professionals should continue to emphasise the importance of limiting added sugars during infancy as part of nutrition education, supported by health promotion campaigns that raise awareness of potential hidden sources of added sugars in complementary foods. Additional support for breastfeeding would also be welcome.

The slightly higher intakes of free sugars and added sugars in severely food insecure infants relative to food secure infants represent just 21 kJ/d of additional energy. Overall, exposure to free and added sugars in the diet was low, as reflected in the 3 % or less of total energy contributed by free sugars or added sugars. Other investigations of added sugars intakes in infants have reported intakes comparable with those in the current study. In the 2009–2014 National Health and Nutrition Examination Survey, added sugars contributed an average of 1·1 % of total energy intake in infants less than 1 year of age, with very small differences of < 0·5 % among levels of household income^(43)^. Likewise, added sugars contributed a median of 2·3 % of daily total energy intake in infants from 6 to 9 months of age in the Dortmund Nutritional and Anthropometric Longitudinally Designed study in Germany^(44)^. Both studies found that the consumption of added sugars increased markedly from 12 months of age, as would be expected since infants are eating family foods by this time.

Notably, our definitions of ‘free sugars’ and ‘added sugars’ are aligned with the Kibblewhite *et al.* ^(28)^ approach used in FOODfiles 2018, but with sugars from fruit purées in wet commercial infant foods not considered to contribute ‘free sugars’ or ‘added sugars’. We considered this appropriate because infant feeding guidelines recommend that fruits are puréed to provide a safe texture as first foods^(45)^. Concerns about the addition of fruit or fruit-derived ingredients to sweeten commercial infant foods led to the WHO Regional Office for Europe’s development of the term ‘liberated sugars’, describing all sugars from commercially puréed fruits^(46)^. However, the argument that intrinsic sugars released from plant cell walls during fruit processing are absorbed into the bloodstream as rapidly as free sugars is not well established. Evidence suggests that sugars in commercial infant fruit purées are not absorbed at a faster rate than sugars in home-prepared purées^(47)^.

This is also relevant to breast milk or infant formula, which contribute most of the energy to the infant diet^(48)^. We did not consider that lactose (an important sugar in breast milk) in infant formula contributed free sugars or added sugars, as its purpose is to provide carbohydrate at levels like those in breast milk. Had our approach not prioritised purpose, the intakes of free and added sugars reported would have been higher, particularly for formula-fed infants and infants consuming large quantities of infant foods containing fruit purées.

On average, BMI z-scores of severely food insecure infants were higher compared with food secure infants, although most infants were of a healthy weight, with no meaningful differences in weight status by household food security status. The prevalence of overweight in our sample was low, and differences between categories were small (with severely food insecure infants having the lowest proportion of overweight). To be able to detect whether differences this small are statistically significant, a much larger sample would be required; however, these results suggest that larger, more meaningful differences in proportions of overweight are unlikely. No studies in the UK or Europe have explored associations between BMI and food insecurity in infants. Studies from the US have reported inconsistent results, with two studies reporting a greater risk of overweight^(15,16)^ and two finding no increased risk^(17,49)^. The lack of agreement between these findings may be, at least in part, a result of differences in the measurement of food insecurity and measures of infant obesity. Moreover, differences between the USA and New Zealand in food availability, social support programs and infant feeding practices may limit the direct comparability of results.

Despite this, potential relationships between food insecurity and infant weight are complex and likely shaped by interrelated factors such as diet quality and the role of food insecurity as a stressor leading to disruptions in caregiving and infant feeding practices^(50)^. Although severely food insecure infants had a higher mean BMI z-score than food secure infants, adjustment for confounding variables strongly attenuated the differences between food insecurity categories. We did not aim to investigate factors influencing both food insecurity and infant BMI, and we recommend that future research shed light on this complex question.

The current study has multiple strengths. A high retention rate was achieved, with two 24-h diet recalls collected from 99 % of participants, which allowed for the calculation of usual nutrient intakes and accurate assessment of breast milk intakes. Another important strength is that infant anthropometric measurements were collected by trained researchers using standardised protocols, rather than being reported by caregivers. Moreover, the measurement of food insecurity at two levels of severity provided a more nuanced understanding of its relationships with infant nutrient intakes and infant BMI.

However, convenience sampling may have introduced sampling bias by attracting participants who were interested in infant nutrition, thus limiting generalisability of the sample to the New Zealand population and those of comparable high-income countries. Nonetheless, the proportion of participants with a higher level of socio-economic deprivation did reflect the proportion in the population as a whole. Second, breast milk intake was estimated in 74 % of infants using an algorithm based on a predictive equation derived from our dose-to-mother subsample, which combined demographic, infant feeding and dietary data^(31)^. While this approach substantially improves the accuracy of nutrition assessment for infants compared with existing methods^(31)^, it may not capture true individual variation. The accuracy of reported food intakes in the 24-h diet recalls could have been influenced by social desirability bias as infant diets were parent reported. In addition, while nutrient intakes were calculated from 2 days of 24-h diet recall data, this may not have captured the infant’s longer-term usual food intake due to within-person variation. However, we tried to minimise this limitation by collecting the 24-h diet recalls on two different days of the week and used the multiple source method to estimate usual intake.

### Conclusions

It is reassuring that differences between the nutrient intakes of infants living in food insecure households and those living in food secure households were small, and that the diets of food insecure infants were likely nutritionally adequate. Overall, this study suggests that, aside from Fe and Zn, New Zealand infants aged 7–10 months are consuming a nutritionally adequate diet regardless of the food security status of the household. Although higher free sugars and added sugars intakes were observed in severely food insecure infants, these differences were small, and the overall exposure to free and added sugars in the diet was low. Finally, no evidence of a relationship between food insecurity and infant overweight was found in this study of 7- to 10-month-olds, with the prevalence of healthy weight high in the sample.

The differences between food insecurity groups observed in this study provide a foundation for examining whether nutrient intakes and BMI differ at other ages, as well as whether these differences in infancy are consistent over time and in other populations. Future research should measure food insecurity over multiple timepoints to understand weight status trajectories in infants and young children living in food insecure households in countries beyond the USA. This understanding could inform early childhood obesity prevention efforts adapted to local contexts to support food insecure families with infants. However, interventions seeking to address household food insecurity must tackle its underlying social and economic drivers. The study also highlights the need to consider the nutrient intakes of all household members, and especially mothers, who may be buffering their children from short-term nutritional impacts and bearing the burden of food insecurity themselves.

## Data Availability

The data used and/or analysed in the present study are not publicly available due to ethical restrictions related to the consent provided by participants. An ethically compliant dataset may be made available by the corresponding author and final author upon reasonable request and upon approval by the Health and Disability Ethics Committee New Zealand.
